# *In Vitro* Activity and Microbiological Efficacy of Gepotidacin from a Phase 2, Randomized, Multicenter, Dose-Ranging Study in Patients with Acute Bacterial Skin and Skin Structure Infections

**DOI:** 10.1128/AAC.01302-19

**Published:** 2020-02-21

**Authors:** Nicole E. Scangarella-Oman, Karen A. Ingraham, Courtney A. Tiffany, Lynn Tomsho, Stephanie F. Van Horn, David N. Mayhew, Caroline R. Perry, Theresa C. Ashton, Etienne F. Dumont, Jianzhong Huang, James R. Brown, Linda A. Miller

**Affiliations:** a Medicine Opportunities Research Unit, GlaxoSmithKline, Collegeville, Pennsylvania, USA; b Target and Pathway Validation, Target Sciences, GlaxoSmithKline Research and Development, GlaxoSmithKline, Collegeville, Pennsylvania, USA; c Computational Biology, Functional Genomics, GlaxoSmithKline Research and Development, GlaxoSmithKline, Collegeville, Pennsylvania, USA; d Computational Biology, Human Genetics, GlaxoSmithKline Research and Development, GlaxoSmithKline, Collegeville, Pennsylvania, USA

**Keywords:** ABSSSI, antibacterial agent, GSK2140944, MRSA, MSSA, *S. aureus*, skin infection, gepotidacin

## Abstract

A phase 2 study of gepotidacin demonstrated the safety and efficacy of 3 gepotidacin doses (750 mg every 12 h [q12h], 1,000 mg q12h, and 1,000 mg every 8 h [q8h]) in hospitalized patients with suspected/confirmed Gram-positive acute bacterial skin and skin structure infections (ABSSSIs). Evaluating microbiology outcomes and responses were secondary endpoints. Pretreatment isolates recovered from infected lesions underwent susceptibility testing per Clinical and Laboratory Standards Institute guidelines. Staphylococcus aureus accounted for 78/102 (76%) of Gram-positive isolates; 54/78 (69%) were methicillin-resistant S. aureus (MRSA), and 24/78 (31%) were methicillin-susceptible S. aureus (MSSA). Posttherapy microbiological success (culture-confirmed eradication of the pretreatment pathogen or presumed eradication based on a clinical outcome of success) for S. aureus was 90% for the gepotidacin 750-mg q12h group, 89% for the 1,000-mg q12h, and 73% in the 1000-mg q8h group. For 78 S. aureus isolates obtained from pretreatment lesions, gepotidacin MIC_50_/MIC_90_ values were 0.25/0.5 μg/ml against both MRSA and MSSA. Isolates recovered from the few patients with posttreatment cultures showed no significant reduction in gepotidacin susceptibility (≥4-fold MIC increase) between pretreatment and posttreatment isolates. Two of the 78 S. aureus isolates from pretreatment lesions had elevated gepotidacin MICs and had mutations known to occur in quinolone-resistant S. aureus (GyrA S84L, ParC S80Y, and ParE D422E) or to confer elevated MICs to novel bacterial topoisomerase inhibitors (GyrA D83N, both isolates; ParC V67A, one isolate). This first report of microbiological outcomes and responses of gepotidacin in patients with ABSSSIs supports further evaluation of gepotidacin as a novel first-in-class antibacterial agent. (This study has been registered at ClinicalTrials.gov under identifier NCT02045797.)

## Abstract (precis)

A phase 2 study of gepotidacin demonstrated the safety and efficacy of 3 gepotidacin doses (750 mg every 12 h [q12h], 1,000 mg q12h, and 1,000 mg every 8 h [q8h]) in hospitalized patients with suspected/confirmed Gram-positive acute bacterial skin and skin structure infections (ABSSSIs). Evaluating microbiology outcomes and responses were secondary endpoints.

## INTRODUCTION

Acute bacterial skin and skin structure infections (ABSSSIs) are the most frequently diagnosed skin infections in both community and hospital settings, and are also associated with substantial morbidity worldwide (1–4). Gram-positive organisms are the predominant pathogens in ABSSSIs, including beta-hemolytic streptococci and Staphylococcus aureus (1, 5–7).

Although most ABSSSIs can be treated on an outpatient basis (8–10), some patients require hospitalization and parenteral antibacterial therapy (1, 6, 11). In the United States between 2005 and 2011, ABSSSIs accounted for 1.8% of all hospital admissions (12). While hospital admission rates for ABSSSIs increased over this time period, mortality rates did not change (12).

Most treatments prescribed for ABSSSIs have been for infections caused by methicillin-susceptible S. aureus (MSSA) and group A streptococci; however, the prevalence of antibiotic-resistant strains, particularly methicillin-resistant S. aureus (MRSA), has significantly increased, and successful treatment with current antibiotics has become increasingly difficult (13). Thus, there is a need for novel antimicrobial agents with unique modes of action that are safe and effective against drug-resistant pathogens.

Gepotidacin (GSK2140944) is a novel, first-in-class triazaacenaphthylene antibiotic that selectively inhibits type IIA topoisomerases through a unique mechanism that is not utilized by any currently approved human therapeutic agent (14). Structural data with a type IIA topoisomerase enzyme, DNA gyrase, revealed the novel binding mode of the triazaacenaphthylene class that is distinct from the binding mode of the quinolone antibacterials (14). Gepotidacin interacts with the bacterial subunits of DNA gyrase (GyrA) and topoisomerase IV (ParC). The stabilized equilibrium state of gepotidacin associates with the uncleaved and single-stranded cleaved DNA complexes to inhibit bacterial DNA replication and cell division (14). Owing to its novel mode of action, *in vitro* studies have shown gepotidacin to be active against most target pathogens resistant to established antibacterials, including fluoroquinolones (14).

O’Riordan et al. (15) reported the efficacy and safety results from a phase 2 study (NCT02045797) that included 122 patients with ABSSSIs given gepotidacin 750 mg or 1,000 mg every 12 h (q12h) or 1,000 mg every 8 h (q8h). The study met the composite primary endpoint of efficacy (early cure rate) and safety (withdrawal rate due to drug-related adverse events) (15). It also demonstrated the potential for gepotidacin as a treatment option for ABSSSIs caused by drug-resistant Gram-positive bacteria. Secondary objectives of this study were to determine the microbiological efficacy of gepotidacin; these results are presented here.

## RESULTS

### Patients and isolates.

The patient demographics and baseline characteristics have been reported previously (15). Of 122 patients in the modified intent-to-treat (mITT) population, 67% (82/122) had at least 1 Gram-positive aerobic pathogen identified from their pretreatment lesion sample and were included in the modified microbiological intent-to-treat (mMITT) population, 18% (15/82) of which had polymicrobial infections.

The majority of the 102 isolates recovered from lesions from the 82 patients in the mMITT population were S. aureus (76% [78/102]). The remaining isolates (24% [24/102]) were other pathogens, including 11% (11/102) other Gram-positive aerobic bacteria (β-hemolytic *Streptococcus* groups A, F, and G, Staphylococcus epidermidis, Staphylococcus lugdunensis, and Streptococcus viridans), 12% (12/102) Gram-negative aerobic bacteria (Acinetobacter baumannii, Acinetobacter species, Enterobacter cloacae, Haemophilus parainfluenzae, Klebsiella pneumoniae, Leclercia adecarboxylata, Pseudomonas aeruginosa, and Serratia marcescens), and 1% (1/102) anaerobic bacteria.

Two patients had positive blood cultures; both were MRSA.

### Susceptibility testing.

Of the 78 S. aureus isolates recovered from pretreatment lesions, 69% (54/78) were MRSA and 31% (24/78) were MSSA. Susceptibility testing demonstrated that 100% of the 78 S. aureus isolates were susceptible to ceftaroline, daptomycin, linezolid, telavancin, tigecycline, and vancomycin regardless of their susceptibility to methicillin. Of these same S. aureus isolates, 62% were resistant to erythromycin, 53% to levofloxacin, 10% to clindamycin, 9% to trimethoprim-sulfamethoxazole, and 5% to tetracycline (Table 1).

**TABLE 1:** Percent resistance for selected antimicrobials against S. aureus isolates from pretreatment lesion samples (mMITT population)^*a*^

Antimicrobial agent	No. (%) of resistant isolates in treatment group: (no. of S. aureus isolates/no. of patients in mMITT population)			Total no. (%) of resistant isolates (no. of S. aureus isolates/no. of patients in mMITT population) (78/82)
	750 mg q12h (39/40)	1,000 mg q12h (28/29)	1,000 mg q8h (11/13)	
Oxacillin	29 (74)	20 (71)	5 (45)	54 (69)
Erythromycin	26 (67)	17 (61)	5 (45)	48 (62)
Levofloxacin	22 (56)	13 (46)	6 (55)	41 (53)
Clindamycin	7 (18)	1 (4)	0 (0)	8 (10)
Trimethoprim-sulfamethoxazole	5 (13)	2 (7)	0 (0)	7 (9)
Tetracycline	2 (5)	2 (7)	0 (0)	4 (5)
Ceftaroline	0 (0)	0 (0)	0 (0)	0 (0)
Chloramphenicol	1 (3)	0 (0)	0 (0)	1 (1)
Gentamicin	1 (3)	0 (0)	0 (0)	1 (1)
Quinupristin/dalfopristin	1 (3)	0 (0)	0 (0)	1 (1)
Daptomycin	0 (0)	0 (0)	0 (0)	0 (0)
Linezolid	0 (0)	0 (0)	0 (0)	0 (0)
Telavancin	0 (0)	0 (0)	0 (0)	0 (0)
Tigecycline	0 (0)	0 (0)	0 (0)	0 (0)
Vancomycin	0 (0)	0 (0)	0 (0)	0 (0)

a The percent resistance was calculated based on the Clinical and Laboratory Standards Institute (CLSI) M100 guidelines. mMITT, modified microbiological intent-to-treat; q8h, every 8 h; q12h, every 12 h.

For the 2 pretreatment MRSA isolates recovered from blood cultures, susceptibility testing demonstrated that both isolates were also resistant to erythromycin and levofloxacin.

### MIC for gepotidacin.

Gepotidacin MIC values against S. aureus isolates from lesion samples are shown in Table 2. Gepotidacin MIC_50_ and MIC_90_ values against the 78 S. aureus isolates recovered from pretreatment lesion samples were 0.25 μg/ml and 0.5 μg/ml, respectively. Gepotidacin MICs ranged from 0.12 to >32 μg/ml against MRSA isolates and 0.12 to 0.5 μg/ml against MSSA isolates.

**TABLE 2:** Gepotidacin MICs against S. aureus isolates recovered from pretreatment lesion samples (mMITT population)^*a*^

Pathogen	No. of isolates	MIC range min to max (μg/ml)	MIC_50_ (μg/ml)	MIC_90_ (μg/ml)
*S. aureus* total	78	0.12 to >32	0.25	0.5
MRSA	54	0.12 to >32	0.25	0.5
MSSA	24	0.12 to 0.5	0.25	0.5

a MIC_50_, median MIC; MIC_90_, 90^th^ percentile MIC; mMITT, modified microbiological intent-to-treat; MRSA, methicillin-resistant S. aureus; MSSA, methicillin-susceptible S. aureus.

**FIG 1:**
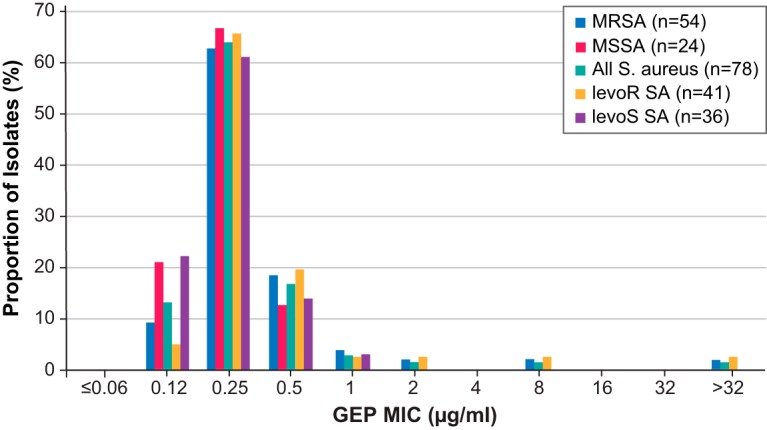
Frequency distribution of gepotidacin MICs against S. aureus isolates from pretreatment lesion samples (mMITT population). GEP, gepotidacin; mMITT, modified microbiological intent-to-treat; MRSA, methicillin-resistant S. aureus; MSSA, methicillin-susceptible S. aureus.

Two S. aureus isolates recovered from pretreatment lesion samples were identified as having an elevated MIC to gepotidacin (MICs >2 μg/ml). One patient had a pretreatment S. aureus isolate with a gepotidacin MIC of 8 μg/ml, and the second patient had a pretreatment S. aureus isolate with a gepotidacin MIC of >32 μg/ml (later determined to be 128 μg/ml). Isolates from both patients were MRSA, and were also resistant to levofloxacin. A second S. aureus isolate was obtained on treatment day 2 from the patient with the pretreatment S. aureus isolate having a gepotidacin MIC of 8 μg/ml. This isolate had the same gepotidacin MIC as the pretreatment isolate and, based on the overall susceptibility profile, was considered to be the same S. aureus strain as the pretreatment isolate. Both patients were treated at the same investigator site. The patient with the pretreatment S. aureus isolate with a gepotidacin MIC of 8 μg/ml was treated prior to the second patient with the pretreatment S. aureus isolate with a gepotidacin MIC of >32 μg/ml. A frequency distribution of gepotidacin MICs against all S. aureus isolates from pretreatment lesion samples is shown in Fig. 1 and frequency distribution of gepotidacin minimum inhibitor concentrations (MICs) against S. aureus isolates from postbaseline lesion samples are in Table S1 in the supplemental material. Both MRSA isolates recovered from blood cultures had a gepotidacin MIC of 0.25 μg/ml. Data for other Gram-positive aerobic pathogens and Gram-negative isolates are shown in Table S2 in the supplemental material.

In this study, there was no evidence of development of resistance to gepotidacin (defined as a ≥4-fold increase in MIC) in isolates collected at any time point (not shown).

### Mutations in S. aureus isolates with elevated gepotidacin MICs.

To understand potential resistance mechanisms, the 3 isolates with elevated gepotidacin MICs from 2 patients were characterized for mutations in the quinolone-resistance determining regions (QRDR) of *gyrA*/*B* and *parC*/*E* genes by PCR and DNA sequencing. All 3 isolates were MRSA and were resistant to ciprofloxacin and levofloxacin. Four target substitutions were identified in all 3 isolates: GyrA S84L, ParC S80Y, and ParE D422E, which were previously known to occur in quinolone-resistant S. aureus isolates (16, 17), and GyrA D83N, a substitution previously known to confer elevated MICs to other novel bacterial topoisomerase inhibitors in laboratory-generated strains (14, 18, 19). An additional substitution, ParC V67A, was found in the patient with a pretreatment S. aureus isolate that had a gepotidacin MIC of >32 μg/ml. Both the GyrA amino acid residue D83 and the ParC amino acid residue V67 are in the novel bacterial topoisomerase inhibitor binding pocket (14).

### Whole-genome sequencing of a subset of S. aureus isolates, including those with elevated gepotidacin MICs.

**FIG 2:**
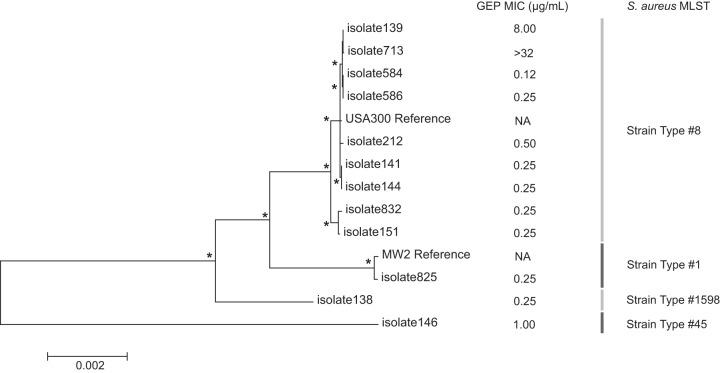
Phylogenetic maximum likelihood tree constructed from a ClustalW alignment of a concatenated set of gene sequences from the 12 isolates in the present study and another 2 reference genomes from GenBank. Nodes of branch points marked with an asterisk were supported in more than 80% of 1,000 bootstrap replications. The scale bar indicates the number of substitutions per position for a unit branch length. Gepotidacin MICs and strain types from MLST analysis appear for each of the sequenced isolates. Gep, gepotidacin; MLST, multilocus sequence typing.

Since all the S. aureus isolates with elevated gepotidacin MICs were from patients treated at the same investigator site, to further understand clonality and epidemiology, 12 S. aureus isolates from that same investigator site (including 2 of the isolates from separate patients with elevated gepotidacin MICs) were phenotypically and temporally selected for whole-genome sequencing (WGS). Multilocus sequence typing (MLST) showed that a majority (9/12) of these isolates belonged to sequence type 8 (ST8) (Table S3 in the supplemental material). Phylogenetic analysis showed a high similarity between the ST8 isolates and the USA300 FPR3757 S. aureus genome (Fig. 2). When comparing the 2 isolates with elevated gepotidacin MICs to the USA300 FPR3757 reference, both isolates shared the GyrA D83N substitution, while isolate 713 (the isolate with a gepotidacin MIC of >32 μg/ml) alone had the ParC V67A substitution. An analysis of all other nonsynonymous variants shared by these 2 isolates (relative to the USA300 FPR3757 reference) identified only 2 other substitutions. The first was a CarA G131S substitution (a carbamoyl-phosphate synthase) and the second was a NirB I416V substitution (a nitrite reductase). This observation suggests that the GyrA D83N and ParC V67A variants are the polymorphisms most relevant to the elevated gepotidacin MICs observed in these isolates.

### Microbiological response and outcome.

The clinical characteristics of the pretreatment lesions have been reported previously, with 44% of patients having a wound infection, 32% a major cutaneous abscess, and 24% cellulitis (15). Microbiological response at the early efficacy visit (day 2 to 3) in lesions infected by S. aureus in the mMITT population after treatment with gepotidacin showed a dose-dependent increase (Table 3). This trend was driven by the larger percentage of S. aureus pathogens with a microbiological outcome of persistence (28%) for the 750 mg q12h treatment group.

**TABLE 3:** Microbiological response and outcomes at the early and posttherapy visits for S. aureus from pretreatment lesion samples (mMITT population)^*a*^

Pathogen microbiological response and outcome	Early efficacy visit				Posttherapy visit			
	750 mg q12h	1,000 mg q12h	1,000 mg q8h	Total	750 mg q12h	1,000 mg q12h	1,000 mg q8h	Total
***S. aureus* total**								
No. patients/no. pathogens	37/39	28/28	11/11	76/78	37/39	28/28	11/11	76/78
No. microbiological success (%) [95% CI]	24 (62) [46.3, 76.8]	19 (68) [50.6, 85.2]	9 (82) [48.2, 97.7]	52 (67) [56.2, 77.1]	35 (90) [75.8, 97.1]	25 (89) [71.8, 97.7]	8 (73) [36.0, 94.0]	68 (87) [79.8, 94.6]
No. eradicated (%)	0	2 (7)	0	2 (3)	3 (8)	1 (4)	0	4 (5)
No. presumed eradicated (%)	24 (62)	17 (61)	9 (82)	50 (64)	32 (82)	24 (86)	8 (73)	64 (82)
No. microbiological failure (%) [95% CI]	15 (38) [23.2, 53.7]	9 (32) [14.8, 49.4]	2 (18) [2.3, 51.8]	26 (33) [22.9, 43.8]	4 (10) [2.9, 24.2]	3 (11) [2.3, 28.2]	3 (27) [6.0, 61.0]	10 (13) [5.4, 20.2]
No. persistent (%)	11 (28)	3 (11)	2 (18)	16 (21)	–	–	–	–
No. presumed persistent (%)	4 (10)	6 (21)	0	10 (13)	3 (8)	2 (7)	1 (9)	6 (8)
No. presumed recurrence (%)	–	–	–	–	1 (3)	1 (4)	2 (18)	4 (5)
**MRSA**								
No. patients/no. pathogens	28/29	20/20	5/5	53/54	28/29	20/20	5/5	53/54
No. microbiological success (%) [95% CI]	18 (62) [44.4, 79.7]	12 (60) [38.5, 81.5]	4 (80) [28.4, 99.5]	34 (63) [50.1, 75.8]	25 (86) [68.3, 96.1]	17 (85) [62.1, 96.8]	4 (80) [28.4, 99.5]	46 (85) [75.7. 94.7]
No. eradicated (%)	0	2 (10)	0	2 (4)	3 (10)	0	0	3 (6)
No. presumed eradicated (%)	18 (62)	10 (50)	4 (80)	32 (59)	22 (76)	17 (85)	4 (80)	43 (80)
No. microbiological failure (%) [95% CI]	11 (38) [20.3, 55.6]	8 (40) [18.5, 61.5]	1 (20) [0.5, 71.6]	20 (37) [24.2, 49.9]	4 (14) [3.9, 31.7]	3 (15) [3.2, 37.9]	1 (20) [0.5, 71.6]	8 (15) [5.3, 24.3]
No. persistent (%)	7 (24)	3 (15)	1 (20)	11 (20)	–	–	–	–
No. presumed persistent (%)	4 (14)	5 (25)	0	9 (17)	3 (10)	2 (10)	0	5 (9)
No. presumed recurrence (%)	–	–	–	–	1 (3)	1 (5)	1 (20)	3 (6)
**MSSA**								
No. patients/no. pathogens	10/10	8/8	6/6	24/24	10/10	8/8	6/6	24/24
No. microbiological success (%) [95% CI]	6 (60) [26.2, 87.8]	7 (88) [47.3, 99.7]	5 (83) [35.9, 99.6]	18 (75) [57.7, 92.3]	10 (100) [69.2, 100.0]	8 (100) [63.1, 100.0]	4 (67) [22.3, 95.7]	22 (92) [73.0, 99.0]
No. eradicated (%)	–	–	–	–	0	1 (13)	0	1 (4)
No. presumed eradicated (%)	6 (60)	7 (88)	5 (83)	18 (75)	10 (100)	7 (88)	4 (67)	21 (88)
No. microbiological failure (%) [95% CI]	4 (40) [12.2, 73.8]	1 (13) [0.3, 52.7]	1 (17) [0.4, 64.1]	6 (25) [7.7, 42.3]	0	0	2 (33) [4.3, 77.7]	2 (8) [1.0, 27.0]
No. persistent (%)	4 (40)	0	1 (17)	5 (21)	–	–	–	–
No. presumed persistent (%)	0	1 (13)	0	1 (4)	0	0	1 (17)	1 (4)
No. presumed recurrence (%)	–	–	–	–	0	0	1 (17)	1 (4)

a CI, confidence interval; mMITT, modified microbiological intent-to-treat; MRSA, methicillin-resistant S. aureus; MSSA, methicillin-susceptible S. aureus; q8h, every 8 h; q12h, every 12 h; –, not applicable.

The patient with the MRSA isolate from the pretreatment lesion sample with a gepotidacin MIC of >32 μg/ml was a clinical and microbiological failure at the early efficacy visit, but clinical and microbiological success was achieved at the posttherapy visit and maintained to the end of the study. The MRSA isolate was the sole pathogen from the infection and was obtained by needle aspiration from an abscess. The patient with the 2 MRSA isolates with the MIC value of 8 μg/ml (from the pretreatment lesion and the treatment day 2 samples) was a clinical and microbiological success at the early efficacy visit, which was maintained throughout the study. The MRSA isolates were the sole pathogens from the infection and were obtained by needle aspiration from an abscess. Per the protocol, for both patients, incision and drainage (I&D) of the lesion was permitted prior to or up to 24 h after the start of the first dose of study medication.

At the posttherapy visit, microbiological success for S. aureus isolates was similar for the 750-mg q12h (90%) and 1,000-mg q12h (89%) treatment groups, but lower for the 1,000-mg q8h treatment group (73%) (Table 3). A similar pattern was observed for the other Gram-positive pathogens (Table S4 in the supplemental material). However, because few posttherapy lesion samples were obtained, the majority of microbiological responses were derived from clinical outcomes and therefore, it is difficult to draw definitive conclusions from these data regarding microbiological response at the posttherapy visit. The small number of patients in the 1,000-mg q8h treatment group also likely contributed to the observed lower success rate.

The 2 patients who had MRSA recovered from their pretreatment blood cultures (both isolates had a gepotidacin MIC of 0.25 μg/ml) were in the 750-mg q12h treatment group and were microbiological successes, with a microbiological outcome of eradication at both the early efficacy and posttherapy visits (not shown).

Definitive conclusions on the role of gepotidacin in the clinical success or failure of patients with Gram-negative isolates could also not be made (Text S1 in the supplemental material).

### Relationship between gepotidacin MIC and microbiological success.

The relationship between gepotidacin MIC and microbiological success against S. aureus isolates from pretreatment lesion samples for each dose group is shown in Table 4. For patients with S. aureus, microbiological success generally increased from the early efficacy visit to the posttherapy visit. However, due to the small number of patients in each MIC category, no definitive conclusions can be drawn from these data regarding the relationship between gepotidacin MIC and microbiological response.

**TABLE 4:** Relationship between gepotidacin MIC and microbiological success against S. aureus isolates from pretreatment lesion samples (mMITT population)^*a*^

Gepotidacin MIC (μg/ml)	No. microbiological success/no. total isolates (%) for:							
	Early efficacy visit				Posttherapy visit			
	750 mg q12h	1,000 mg q12h	1,000 mg q8h	Total	750 mg q12h	1,000 mg q12h	1,000 mg q8h	Total
≤0.06	–	–	–	–	–	–	–	–
0.12	1/3 (33)	4/6 (67)	1/1 (100)	6/10 (60)	3/3 (100)	5/6 (83)	1/1 (100)	9/10 (90)
0.25	15/27 (56)	9/13 (69)	8/10 (80)	32/50 (64)	23/27 (85)	12/13 (92)	7/10 (70)	42/50 (84)
0.5	5/6 (83)	6/7 (86)	–	11/13 (85)	6/6 (100)	6/7 (86)	–	12/13 (92)
1	1/1 (100)	0/1 (0)	–	1/2 (50)	1/1 (100)	1/1 (100)	–	2/2 (100)
2	1/1 (100)	–	–	1/1 (100)	1/1 (100)	–	–	1/1 (100)
4	–	–	–	–	–	–	–	–
8	1/1 (100)	–	–	1/1 (100)	1/1 (100)		–	1/1 (100)
16	–	–	–	–	–	–	–	–
32	–	–	–	–	–	–	–	–
>32	–	0/1 (0)	–	0/1 (0)	–	1/1 (100)	–	1/1 (100)

a mMITT, modified microbiological intent-to-treat; q8h, every 8 h; q12h, every 12 h; –, no results applicable.

## DISCUSSION

The majority (76% [78/102]) of isolates recovered from patients in the modified microbiological intent-to-treat (mMITT) population were S. aureus, and 69% (54/78) of the S. aureus isolates from lesions were MRSA. Additionally, 2 isolates recovered from blood cultures were MRSA.

Gepotidacin had MIC_50_ and MIC_90_ values of 0.25 μg/ml and 0.5 μg/ml, respectively, for both MRSA and MSSA isolates recovered from pretreatment lesion samples. Microbiological success was achieved at the early efficacy visit in most patients, and showed a dose-dependent increase driven by the larger percentage of S. aureus pathogens with a microbiological outcome of persistence for the 750-mg q12h treatment group. Based on available pharmacokinetic/pharmacodynamic (PK/PD) data for gepotidacin, these success percentages were not unexpected (15, 20). At the posttherapy visit, microbiological success was lowest at the highest dose studied, 1,000 mg q8h. This was likely driven by the small number of patients in this treatment group, as well as all microbiological responses being derived from clinical outcomes due to the lack of posttherapy lesion samples that were obtained at this visit.

While reports of the impact on gepotidacin activity and efficacy against an analogous mutation, ParC D86N, in clinical isolates of Neisseria gonorrhoeae have recently been reported (21, 22), to our knowledge, this is the first report of clinical S. aureus isolates with elevated gepotidacin MICs or mutations in GyrA D83 or ParC V67. A previous study reported that the highest gepotidacin MIC seen for a global collection of >1,000 S. aureus isolates was 2 μg/ml (23). In this study, both pretreatment S. aureus isolates with elevated gepotidacin MICs were recovered from patients at a single investigator site. Given that mutations in GyrA D83 or ParC V67 have not been shown to preexist in clinical S. aureus isolates, one potential hypothesis is that the first step, a GyrA D83N mutation, occurred as a result of selection pressure from the gepotidacin treatment of an earlier patient at this investigator site, which then resulted in infection by this strain of the patient from which the first S. aureus isolate with an elevated gepotidacin MIC was recovered. The selection pressure from treatment of this patient could have then caused the second step mutation, ParC V67A, which was recovered after subsequent infection of the later patient.

The phylogenetic analysis of the WGS sequencing data showed that both of these isolates were highly related and supports the hypothesis that one isolate emerged from the other and that the GyrA D83N mutations did not arise independently. Although we were not able to obtain evidence to specifically prove this hypothesis, it is plausible given the behaviors of some patients in this study (e.g., intravenous drug use and sharing of needles) (15).

There are a few limitations to this study that should be considered. No comparator agent was used for treatment in this study. Thus, it is not possible to draw conclusions regarding comparisons of the microbiological efficacy of gepotidacin with other antimicrobial agents. In this study, 44% of patients had a wound infection, while 32% had a major cutaneous abscess and 24% had cellulitis. In contrast, registration studies of ABSSSIs for other recently approved antibacterial agents had patient populations with an infection type distribution that ranged from approximately 40% to 50% with cellulitis or erysipelas, 20% to 31% with a major cutaneous abscess, and 20% to 30% with a wound infection (24–26). Additionally, it was generally not possible to get posttherapy specimens because the wound had healed; therefore, the microbiological outcomes and responses at posttreatment visits were primarily based on the clinical outcomes and responses. Assessment of failures by infection type, organism, MIC, etc., was limited by the small number of posttherapy specimens available. Additionally, due to the small sample size in this phase 2 study, analysis comparing mono- and polymicrobial infections was not conducted. This analysis and additional characterization of pre- and posttherapy isolates would be necessary in later stage clinical trials for registration. Finally, this study was limited by geographic distribution because it was conducted exclusively at study centers in the United States.

In conclusion, against the 78 S. aureus isolates recovered from pretreatment lesions, gepotidacin MIC_50_ and MIC_90_ values were 0.25 μg/ml and 0.5 μg/ml, respectively, for both MRSA and MSSA. S. aureus isolates with elevated gepotidacin MICs (>2 μg/ml) were recovered from lesion samples prior to treatment in 2 patients. These isolates had mutations that were in the gepotidacin-binding pocket. There was no evidence of on-therapy or posttherapy development of resistance to gepotidacin. From the limited data available, at the early efficacy visit, a positive dose-response relationship appeared to be present for increasing gepotidacin doses and microbiological response, with the greatest microbiological success observed in the gepotidacin 1,000-mg q8h treatment group. This first report of gepotidacin microbiological efficacy in the treatment of patients with ABSSSI supports further clinical study of gepotidacin as a novel, first-in-class antibacterial agent.

## MATERIALS AND METHODS

### Patients.

The study (NCT02045797 available at ClinicalTrials.gov) included patients ≥18 years of age with a suspected or confirmed Gram-positive acute bacterial skin and skin structure infection (ABSSSI) involving a wound infection characterized by purulent drainage with surrounding redness, edema, and/or an induration with a minimum surface area of 75 cm^2^; a major cutaneous abscess characterized by a collection of pus accompanied by redness, edema, and/or an induration with a minimum surface area of 75 cm^2^; or cellulitis with an induration with a minimum surface area of 75 cm^2^. The inclusion criteria have been reported previously (15).

The modified intent-to-treat (mITT) population consisted of all randomly assigned patients who received at least 1 dose of study medication. This population was the primary analysis population for the safety and efficacy analyses.

The modified microbiological intent-to-treat (mMITT) population consisted of all randomly assigned patients who received at least 1 dose of study medication and had a Gram-positive aerobic pathogen identified from their pretreatment bacteriology lesion sample. This was the primary analysis population for microbiological endpoint analyses.

### Ethical approval.

Written, informed consent was obtained from all patients, and the study was conducted in accordance with good clinical practice as defined by the International Council for Harmonization and the Declaration of Helsinki 2008 (15). The protocol, amendments, and patient-informed consent were approved by a local or academic institutional review board prior to initiation of the study.

### Study design.

Data were collected during a phase 2, randomized, multicenter, dose-ranging, Bayesian response-adaptive study that was conducted in the United States (NCT02045797). The study design has been described in detail previously (15). Briefly, patients were treated with 1 of 3 intravenous (IV) gepotidacin doses as follows: 750 mg q12h, 1,000 mg q12h, or 1,000 mg q8h. Part 1 was initiated with double-blind IV treatment for a minimum of 2 days and a maximum of 10 days in an inpatient setting. At the discretion of the investigator, patients who completed the minimum IV dosing duration of 2 days could be switched to a corresponding open-label oral dosing regimen in an outpatient setting in part 2 to complete the total 10 days of treatment.

### Microbiological assessments.

Lesion samples were obtained by tissue biopsy specimen, needle aspiration, or skin swab from all patients at the pretreatment and at all posttreatment visits where culturable material was present. A swab sample was only obtained when there was sufficient pus or exudate to heavily impregnate the swab and, in the opinion of the investigator, collection by biopsy or aspiration was not appropriate. Blood sampling was done prior to treatment, at any posttherapy visits, and when patients were deemed a clinical failure or exhibiting signs/symptoms of bacteremia or sepsis. All positive blood cultures were repeated until the sample tested negative for infection. All lesion and blood samples were sent to a local laboratory for Gram stain, culture, and pathogen identification. All protocol-defined pathogens were then sent to a central laboratory for confirmatory bacterial identification and susceptibility testing on all Gram-positive aerobic pathogens.

Microbiological success was defined as culture-confirmed eradication of the pretreatment pathogen or was derived from clinical outcome of success in the absence of a posttreatment specimen, and was determined at the early efficacy (day 2 to 3) and posttreatment (day 12 to 18) visits.

### Susceptibility.

Susceptibility was tested at a central laboratory by broth microdilution, according to Clinical and Laboratory Standards Institute (CLSI) guidelines. Tests for detection of β-lactamase production and inducible clindamycin resistance were also conducted according to CLSI guidelines. Development of reduction in susceptibility to gepotidacin in pathogens was evaluated by comparing pretreatment gepotidacin MIC values for Gram-positive aerobic isolates with any values obtained posttreatment for the same pathogen. Gram-positive aerobic isolates obtained posttreatment from the same patient with a confirmed ≥4-fold increase in gepotidacin MIC (μg/ml) for the same pathogen, were considered to have developed a reduction in susceptibility to gepotidacin.

### QRDR genotyping.

Extended quinolone-resistance determining regions (QRDR) for *gyrA*, *gyrB*, *parC*, and *parE* were PCR amplified and sequenced to identify mutations resulting in amino acid substitutions (27). The following QRDR oligonucleotide primers were used:

*gyrA*: 895 bp product

Primer1: 5′-CGTTGTAGAAAACCGTAGACA-3′

Primer2: 5′-GGAATTTCAGTGACAACA-3′

*gyrB*: 910 bp product

Primer1: 5′-CAAACATGGTGATCCTCA-3′

Primer2: 5′-GGTGTTGGATTCAATTCAGA-3′

*parC*: 730 bp product

Primer1: 5′-GAGTTTGGTATGCAAGAGGACC-3′

Primer2: 5′-CCTTTACCTGATTCATAAGC-3′

*parE*: 630 bp product

Primer1: 5′-CGTGAAGGTTTAACAGCTGTTGTG-3′

Primer2: 5′-CTCTTCGTCTGTCCAAGC-3′

PCR products were purified using a QIAquick PCR purification kit (Qiagen Sciences, Inc., Germantown, MD) and sequenced using the AB v3 1 BigDye-terminator cycle sequencing kit (Applied Biosystems). Sequence alignments were carried out using Lasergene MegAlign software (DNASTAR, Inc., Madison, WI) to identify mutations resulting in amino acid residue substitutions.

### Illumina library preparation and WGS.

DNA samples were prepared from monocultures of single bacterial colonies using Promega Maxwell 16 cell DNA purification kit and instrument (Promega Corporation, Madison, WI). DNA samples were quantitated using Qubit fluorometric double-stranded DNA (dsDNA) broad-range (BR) kit and instrumentation (Thermo Fisher Scientific, Waltham, MA). Illumina libraries (average library insert size, 350 bp) were constructed using Illumina TruSeq Nano DNA library prep kit B (Illumina, Inc., San Diego, CA) with some modifications. Genomic DNA (1 μg in 50 μl Tris-EDTA [TE] buffer × 2 for a total of 2 μg) was sheared for 180 s (Duty Factor 10, Peak Power 175, 200 cycles per burst) on a Covaris S220 sonicator (Covaris, Inc., Woburn, MA). Libraries were enriched with 6 cycles of PCR following protocol-cycling conditions. Fragment libraries were size selected (300 to 400 bp insert range), and primer dimers were removed using Agencourt AMPure XP bead purification (Beckman Coulter, Inc., Brea, CA). All libraries were checked for proper fragment size using Agilent High Sensitivity DNA kit on an Agilent 2100 Bioanalyzer (Agilent Technologies, Inc., Santa Clara, CA). For accurate quantification, quantitative PCR (qPCR) was performed in triplicate using a KAPA library quantification kit (Kapa Biosystems, Wilmington, MA) on the 7900HT real-time PCR instrument (Life Technologies, Applied Biosystems, Foster City, CA). Samples (10 nM) were normalized and combined into 1 pool. A unique adapter index sequence was used for each sample to allow for independent samples to be pooled for sequencing and subsequent bioinformatic segregation of the data output. Libraries were diluted to a final 17 pmol dilution. The Illumina MiSeq sequencer instrument (Illumina, Inc., San Diego, CA) reagents and flow cell were prepared according to Illumina MiSeq v3 protocols. A MiSeq 50-cycle run was conducted to check proper cluster density and library normalization. A full MiSeq 600-cycle run (2 × 300 bp) was run to collect the sequence data.

### Sequence and Phylogenetic Analysis.

Trimming of the unaligned FASTQ files was performed using Trimmomatic v0.33 (28). MLST was performed using reads aligning to the 7 housekeeping genes *arcC*, *aroE*, *glpF*, *gmk*, *pta*, *tpi*, and *yqil* and sequence types (STs) were determined using the S. aureus multilocus sequence typing (MLST) database (http://saureus.mlst.net/) (29). To identify nonsynonymous variants in all annotated genes, trimmed MiSeq reads were then aligned to the closest reference genome as a scaffold: USA300-FPR3757 (NC_007793.1), MW2 (NC_003923.1), or MRSA252 (NC_002952.2) using Breseq v0.26.1 (30) at a mean coverage of 160 reads per base. Phylogenetic relationships were determined from a concatenated sequence file consisting of 53,026 nucleotides from gene sequences aligned by ClustalW. A neighbor-joining tree with 1,000 bootstrap replicates was reconstructed using the PHYLIP (v3.6) package (31). Phylogenetic tree figures were generated with the software MEGA v6 (32).

### Data availability.

Within 6 months of this publication, anonymized individual participant data, the annotated case report form, protocol, reporting and analysis plan, data set specifications, raw data set, analysis-ready data set, and clinical study report will be available for research proposals approved by an independent review committee. Proposals should be submitted to www.clinicalstudydatarequest.com. A data access agreement will be required.

## Supplementary Material

Supplemental file 1
