# Supplementary material for: In Vitro Activity and Microbiological Efficacy of Gepotidacin from a Phase 2, Randomized, Multicenter, Dose-Ranging Study in Patients with Acute Bacterial Skin and Skin Structure Infections
Source: Antimicrob Agents Chemother. 2020 Feb 21;64(3):e01302-19. doi: 10.1128/AAC.01302-19 (PMC7038298; doi:10.1128/AAC.01302-19)
Supplement: Supplemental file 1 [file AAC.01302-19-s0001.pdf]

Supplemental Materials

TABLE S1. Frequency distribution of gepotidacin MICs **(µg/ml)** against *S. aureus* from lesion samples by visit (mMITT population).

**Visit (n)**	**Number of isolates with indicated gepotidacin MIC**											
		**≤ 0.06**	**0.12**	**0.25**	**0.5**	**1**	**2**	**4**	**8**	**16**	**32**	**>32**
Baseline (78)		0	10	50	13	2	1	0	1	0	0	1
IV – Day 1 (3)		0	0	3	0	0	0	0	0	0	0	0
IV – Day 2 (30)		0	5	19	5	0	0	0	1	0	0	0
Early Efficacy (17)		0	4	12	1	0	0	0	0	0	0	0
IV – Day 3 (4)		0	0	4	0	0	0	0	0	0	0	0
IV – Day 4 (1)		0	0	1	0	0	0	0	0	0	0	0
IV – Day 5 (3)		0	0	3	0	0	0	0	0	0	0	0
IV – Day 6 (2)		0	0	2	0	0	0	0	0	0	0	0
IV – Day 7 (1)		0	0	1	0	0	0	0	0	0	0	0
IV – Day 8 (1)		0	0	1	0	0	0	0	0	0	0	0
Oral – Day 1 (3)		0	0	3	0	0	0	0	0	0	0	0
Oral – Day 7-10 (1)		0	0	0	1	0	0	0	0	0	0	0
Overall Postbaseline (66)		0	9	49	7	0	0	0	1	0	0	0

IV, intravenous; MIC, minimum inhibitory concentration; mMITT, modified microbiological intent-to-treat.

TABLE S2. Gepotidacin MICs against Gram-positive (other than S. *aureus*) and Gram-negative aerobic isolates recovered from pretreatment lesion samples (mMITT population).

**Pathogen**	**Number of isolates**	**MIC range, min – max (µg/ml)**	**MIC_{50} (µg/ml)**	**MIC_{90} (µg/ml)**
**Gram-positive aerobic pathogens (excluding *S. aureus*)[a]**	11	≤0.06 to 0.5	0.25	0.5
**Gram-negative aerobic isolates**				
Klebsiella pneumoniae	8	4 to 8	8	8
Serratia marcescens	3	8	NA	NA
Escherichia coli	1	4	NA	NA
Enterobacter cloacae	2	8 to 16	NA	NA
Leclercia adecarboxylata	1	4	NA	NA
Acinetobacter spp.	1	32	NA	NA
Pseudomonas aeruginosa	2	8	NA	NA
Haemophilus spp.	2	2	NA	NA
Klebsiella oxytoca	1	2	NA	NA

MIC, minimum inhibitory concentration; MIC_{50}, median minimum inhibitory concentration; MIC_{90}, 90th percentile minimum inhibitory concentration; mMITT, modified microbiological intent-to-treat.

[a]Other Gram-positive aerobes included β-hemolytic *Streptococcus* groups A, F, and G; *Staphylococcus epidermidis*; *Staphylococcus lugdunensis;* and *Streptococcus viridans.*

TABLE S3. Multilocus sequence typing (MLST) of the 12 isolates selected for whole genome sequencing using 7 housekeeping genes in the *S. aureus* MLST database.

SampleID	*arcC*	*aroE*	*glpF*	*gmk*	*pta*	*tpi*	*yqil*	MLST Strain Type	Gepotidacin MIC (µg/mL)
isolate138	12	1	37	15	11	1	40	1598	0.25
isolate139	3	3	1	1	4	4	3	8	8
isolate141	3	3	1	1	4	4	3	8	0.25
isolate144	3	3	1	1	4	4	3	8	0.25
isolate146	10	14	8	6	10	3	2	45	1
isolate151	3	3	1	1	4	4	3	8	0.25
isolate212	3	3	1	1	4	4	3	8	0.5
isolate584	3	3	1	1	4	4	3	8	0.12
isolate586	3	3	1	1	4	4	3	8	0.25
isolate713	3	3	1	1	4	4	3	8	>32
isolate825	1	1	1	1	1	1	1	1	0.25
isolate832	3	3	1	1	4	4	3	8	0.25

MIC, minimum inhibitory concentration; MLST, multilocus sequence typing.

TABLE S4. Microbiological response and outcomes at the early and posttherapy visits for Gram-positive pathogens (other than *S. aureus)* from pretreatment lesion samples (mMITT population).

**Pathogen** **Microbiological response and outcome**	**Early efficacy visit**				**Posttherapy visit**			
	**750 mg q12h**	**1,000 mg q12h**	**1,000 mg q8h**	**Total**	**750 mg q12h**	**1,000 mg q12h**	**1,000 mg q8h**	**Total**
**Other Gram-positive pathogens**								
Patients/pathogens, n/n	4/4	4/4	3/3	11/11	4/4	4/4	3/3	11/11
Microbiological success, n (%) [95% CI]	2 (50) [6.8, 93.2]	3 (75) [19.4, 99.4]	3 (100) [29.2, 100.0]	8 (73) [39.0, 94.0]	3 (75) [19.4, 99.4]	3 (75) [19.4, 99.4]	2 (67) [9.4, 99.2]	8 (73) [39.0, 94.0]
Eradication	2 (50)	2 (50)	0	4 (36)	-	-	-	-
Presumed eradication	0	1 (25)	3 (100)	4 (36)	3 (75)	3 (75)	2 (67)	8 (73)
Microbiological failure, n (%) [95% CI]	2 (50) [6.8, 93.2]	1 (25) [0.6, 80.6]	0	3 (27) [6.0, 61.0]	1 (25) [0.6-80.6]	1 (25) [0.6-80.6]	1 (33) [0.8-90.6]	3 (27) [6.0-61.0]
Persistence	1 (25)	1 (25)	0	2 (18)	–	–	–	–
Presumed persistence	1 (25)	0	0	1 (9)	0	1 (25)	0	1 (9)
Presumed recurrence	–	–	–	–	1 (25)	0	1 (33)	2 (18)

CI, confidence interval; mMITT, modified microbiological intent-to-treat; q8h, every 8 h; q12h, every 12 h.

Text S1. Clinical success/failure in Gram-negative isolates

Definitive conclusions on the role of gepotidacin in the clinical success or failure of patients with Gram-negative isolates could not be made. Of the 17 patients in the mITT population who had a Gram-negative aerobic isolate recovered from their baseline lesion specimen, only 3 who had a Gram-negative aerobic pathogen did not receive concomitant antibacterial therapy and also did not have a co-infecting pathogen. These patients included 1 patient in the 750 mg q12h treatment group with an *Escherichia coli* isolate with a gepotidacin MIC value of 4 µg/ml; 1 in the 1000 mg q12h treatment group with a *K. pneumoniae* isolate with a gepotidacin MIC value of 4 to 8 µg/ml; and 1 in the 1000 mg q8h treatment group with a *Haemophilus* spp. isolate with a gepotidacin MIC value of 2 µg/ml.
